# Wild-Type *Drosophila melanogaster* as a Model Host to Analyze Nitrogen Source Dependent Virulence of *Candida albicans*

**DOI:** 10.1371/journal.pone.0027434

**Published:** 2011-11-14

**Authors:** Monica M. Davis, Francisco J. Alvarez, Kicki Ryman, Åsa A. Holm, Per O. Ljungdahl, Ylva Engström

**Affiliations:** 1 Department of Molecular Biology and Functional Genomics, Stockholm University, Stockholm, Sweden; 2 Wenner-Gren Institute, Stockholm University, Stockholm, Sweden; Alexander Flemming Biomedical Sciences Research Center, Greece

## Abstract

The fungal pathogen *Candida albicans* is a common cause of opportunistic infections in humans. We report that wild-type *Drosophila melanogaster* (OrR) flies are susceptible to virulent *C. albicans* infections and have established experimental conditions that enable OrR flies to serve as model hosts for studying *C. albicans* virulence. After injection into the thorax, wild-type *C. albicans* cells disseminate and invade tissues throughout the fly, leading to lethality. Similar to results obtained monitoring systemic infections in mice, well-characterized *cph1Δ efg1Δ* and *csh3Δ* fungal mutants exhibit attenuated virulence in flies. Using the OrR fly host model, we assessed the virulence of *C. albicans* strains individually lacking functional components of the SPS sensing pathway. In response to extracellular amino acids, the plasma membrane localized SPS-sensor (Ssy1, Ptr3, and Ssy5) activates two transcription factors (Stp1 and Stp2) to differentially control two distinct modes of nitrogen acquisition (host protein catabolism and amino acid uptake, respectively). Our results indicate that a functional SPS-sensor and Stp1 controlled genes required for host protein catabolism and utilization, including the major secreted aspartyl protease *SAP2*, are required to establish virulent infections. By contrast, Stp2, which activates genes required for amino acid uptake, is dispensable for virulence. These results indicate that nutrient availability within infected hosts directly influences *C. albicans* virulence.

## Introduction

Largely due to growing numbers of immune-compromised individuals, fungal infections in humans are becoming an increasing concern [1], [2], [3]. *Candida albicans* is principally responsible for the increased incidence of fungal infections, and is currently the fourth most common cause of septicemia in developed countries [4], [5], [6]. The efficacy of current treatment regimes is being challenged by emerging drug resistance, and antifungal drugs often manifest severe and undesirable side-effects [7]. Information regarding basic fungal biology and virulence traits is critical to facilitate the development of novel treatment strategies.

Like all microorganisms, *C. albicans* relies on its capacity to take up nutrients from the environment, and consequently, many fungal-specific gene products involved in nutrient transport are expected to be essential during virulent growth. In contrast to many microbial pathogens, *C. albicans* has a diverse metabolic repertoire and is able to colonize virtually any tissue and organ [8], [9], where it grows in yeast-like, pseduohyphal and hyphal forms; however, little is known regarding what nutrients are actually utilized during infectious growth. A required nutrient is nitrogen, which is readily available in two forms in infected hosts, amino acids and proteins. *C. albicans* cells possess the means to utilize both of these forms of nitrogen [10].

**Figure 1:**
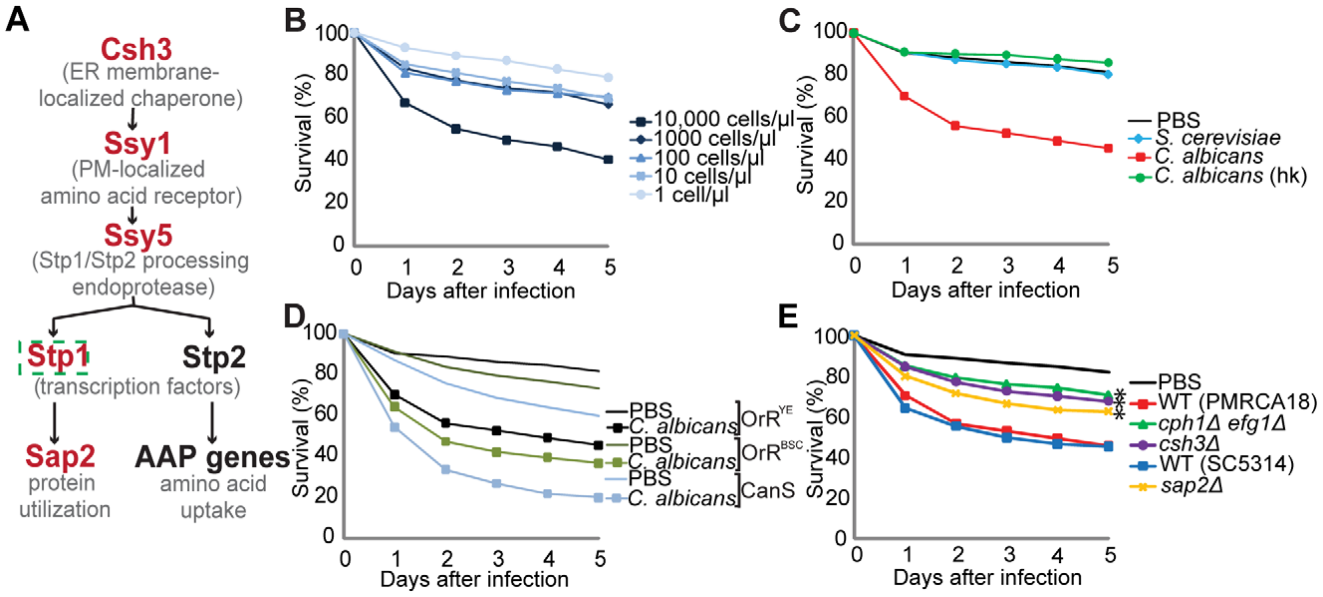
Drosophila can be used as a model of *C. albicans* virulence. A. The epistatic relationships of the components of the SPS sensing pathway in *C. albicans*. Strains carrying null alleles of the genes in red type exhibit reduced virulence in the *Drosophila* host model. The dashed-line around *STP1* indicates that this gene is nitrogen regulated, and is only expressed under conditions of limiting nitrogen-source availability. Stp1 activates genes involved in host protein degradation and utilization, such as the gene encoding the Sap2 protease. Stp2 activates genes required for amino acid uptake, such as genes for amino acid permeases (AAPs). Note that Ssy1 is identical to Csy1 [14]; Ssy1 is used here since it is the standard name listed in the Candida Genome Database. B. Wild-type Drosophila (OrR^YE^) flies are susceptible to virulent *C. albicans* infections and can be used as a model host to assess the virulence properties of mutant *C. albicans* strains. Ten-fold serial dilutions (ranging from 10,000 cells/µl to 1 cell/µl as indicated) of *C. albicans* (PMRCA18) exhibit dose-dependent lethality in OrR flies (n = 500). C. OrR flies (n = 500) were injected with PBS, *S. cerevisiae* (KRY001), viable or heat-killed (hk) *C. albicans* (WT). Fungal cells were suspended at a concentration of 10,000 cells/µl. D. Different wild-type *Drosophila* lines, OrR^YE^, a newly obtained OrR line (OrR^BSC^) and CantonS, were injected with PBS or *C. albicans* (WT; PMRCA18) at 10,000 cells/µl. E. Flies (n = 500) were injected with PBS, *C. albicans* WT (PMRCA18 or SC5314), *cph1Δ efg1Δ*, *csh3Δ*, or *sap2Δ* suspended at 10,000 cells/µl. Statistically significant differences from the survival of the flies injected with WT *C. albicans* are marked by an asterisk.

*C. albicans* utilizes the SPS sensing pathway (see Figure 1A for a schematized summary) to coordinate nitrogen source utilization [10]. The SPS sensing pathway was first identified in the yeast *Saccharomyces cerevisiae* (reviewed in [11], and derives its name from the SPS-sensor, a plasma membrane-localized trimeric receptor complex comprised of three core components, i.e., **S**sy1, **P**tr3 and **S**sy5 [12]. The *C. albicans* genome encodes homologues of all characterized SPS sensing pathway components [13], and available data suggest that these components function similarly to their *S. cerevisiae* counterparts [10], [13], [14]. Ssy1 is the primary amino acid receptor [14], [15], Ptr3 apparently functions as a scaffold protein required to properly control Ssy5 [16], [17], and Ssy5 is a signaling endoprotease [16], [17], [18], [19], [20]. Stp1 and Stp2 are transcription factors that are synthesized as latent cytoplasmic proteins [10], [21]. In response to µM concentrations of extracellular amino acids, and in a strictly SPS-sensor dependent manner, Stp1 and Stp2 are cleaved by Ssy5. The shorter forms of Stp1 and Stp2 efficiently translocate into the nucleus where they induce the expression of SPS-sensor controlled genes [10], [21], [22].

In *C. albicans*, processed Stp1 activates the expression of genes encoding proteins required for the catabolic utilization of extracellular proteins, including the secreted aspartyl protease *SAP2* [10], [23]. Processed Stp2 induces the expression of several amino acid permease genes (AAPs), encoding the proteins that transport amino acids into cells [10]. *SAP2* is required for *C. albicans* virulence in various mammalian hosts [24], [25], [26], [27]. The finding that SPS-sensor activation of Stp1 is required for *SAP2* expression indicates that nutrient-induced signals regulate important virulence factors.

The most upstream component of the *Candida* SPS sensing pathway is Csh3, an ER membrane-localized chaperone that is required for the proper localization of AAPs and Ssy1 to the plasma membrane of *C. albicans* cells [13], [28]. Consequently, *csh3* null mutants lack a functional SPS sensing pathway and exhibit a greatly diminished capacity to take up amino acids, and do not undergo morphological transitions in response to inducing amino acids [13]. Cells bearing a genomic deletion of *csh3* exhibit attenuated virulence compared to wild-type following injection into mice. This demonstrates the importance of nitrogen assimilation to *C. albicans* virulence and suggests that fungal cells require the capacity to respond to amino acids for growth in mammalian hosts [13].

Following the completion of the *C. albicans* genome sequence [29], systematic efforts to create a complete set of null alleles have been pursued, e.g., [30]. Mammalian models are undoubtedly important for virulence assays; however, primary scans of extensive mutant collections would benefit from using alternative host models, which could decrease any financial, logistical, and ethical concerns with mammalian models. Since adaptive immunity is dispensable for host defence against invasive *Candida* infection in mice [31], *Drosophila melanogaster*, which elicits only an innate immune response, is well suited as a mini-host model. *Drosophila* is a well-established and advanced model for the studies of host-pathogen interactions [32]. Studies examining fungal immunity in *Drosophila* have shown that the response to these infections is managed through the activation of the Toll pathway via the Toll receptor (encoded by *Tl*) [33]. Intracellular signal transduction activates Toll responsive immune gene expression including the gene encoding the anti-fungal peptide Drosomycin [34], [35].

Previous work examining *C. albicans* virulence in flies has relied exclusively on the use of mutant strains of *Drosophila* lacking Toll pathway function [36], [37], [38]. Unfortunately, the use of these mutants has introduced experimental limitations that have compromised the usefulness of this mini-host system. In particular, the Toll pathway mutants are severely immuno-compromised and thus inadequate for the analysis of all but the most severely compromised fungal strains.

We report that wild-type *Drosophila* stocks, such as the common laboratory strain, OregonR (OrR), are suitable to study *C. albicans* virulence. After infection via injection into the thorax, *C. albicans* cells are found disseminated throughout the fly and many morphological forms are present. Following an initial acute stage of infection, lasting a period of three days, an apparent balance is reached in the host-pathogen interaction resulting in a persistent infection. We use this insect host model to examine the importance of SPS sensing pathway components in promoting virulent infection, and show that Stp1, which specifically activates genes required for the catabolic utilization of host proteins, including *SAP2*, is required for full virulence. Mutations affecting signaling components upstream of Stp1, *i.e.,* the plasma membrane-localized amino acid receptor Ssy1 and the Stp1 processing endoprotease Ssy5, also show reduced virulence. By contrast, deletion of Stp2, which activates genes required for amino acid uptake, did not impair virulence. These results clearly demonstrate the suitability of using wild-type *D. melanogaster* to study *C. albicans* virulence.

## Results

### Wild-type *Drosophila* as a model for *C. albicans* virulence

The adaptive immune response appears to be of limited importance for host defense against invasive *Candida* infections in mice [31]. Consequently, *D. melanogaster*, which depend exclusively on an innate immune response to protect against pathogens, have been considered appropriate models to study *Candida* infections [36], [37], [38]. We have pursued this notion and envisioned that a refinement of the *Drosophila* model could provide a robust assay system to assess *C. albicans* virulence. We set up the following criteria: 1) high sensitivity to allow visualization of subtle differences in virulence – this required that we avoid Toll pathway mutant flies; 2) simple infection strategy – we opted to use a standard injection system with well-established protocols to introduce reproducible quantities of fungal cells into individual flies; and 3) clear and unambiguous read-out – the virulence assessment should be simple, quick, and require no specialized training in *Drosophila* genetics.

To determine if wild-type *Drosophila* could be used for *C. albicans* virulence studies, the common laboratory strain OrR was injected with different concentrations of wild-type *C. albicans* cells and survival was compared to those flies injected with PBS (Figure 1B and Table S1). A concentration of 10,000 cells/µl (approximately 500 cells/fly) is sufficient to induce significant lethality (p-value, day 3<0.001). We noted that lethality was dependent on the use of *C. albicans* cells grown to log-phase (OD_600_ ≈ 1); flies are less susceptible to infection when stationary phase cells are injected (data not shown). Infection with concentrations of 1000 cells/µl, 100 cells/µl, or 10 cells/µl induced moderate lethality (p-values, day 3 = 0.007, 0.005, and 0.029, respectively), while a concentration of 1 cell/µl failed to kill the flies (p-value, day 3 = 0.886). Based on these results, a concentration of 10,000 cells/µl was chosen for all subsequent experiments.

We sought confirmation that fly lethality was a consequence of *bona fide* virulent properties of this fungal pathogen and to determine whether OrR flies could be used to establish a *C. albicans* virulence assay. For all experiments displayed in Figures 1E, 4 and S1, double-blind experiments were performed, and Table S3 summarizes the pair-wise statistical analysis of lethality at day 3 post-infection. Viable wild-type *C. albicans* caused significant lethality, while heat-killed preparations of the same strain showed no virulence (Figure 1C). Since living cells are required, lethality is not a consequence of a toxic-shock response. Next we examined whether a related, but non-pathogenic fungal species, could induce lethality. For this purpose, we constructed a diploid prototrophic *S. cerevisiae* strain derived from the Σ1278b background. Similar to *C. albicans*, Σ1278b-derived diploid strains undergo controlled morphological transitions, i.e., from unpolarized non-filamentous to filamentous pseudohyphal growth [39], and, thus, represent better controls than the often used haploid S288c background strains. Flies injected with *S. cerevisiae* were asymptomatic and survived the infection as well as the PBS controls (Figure 1C).

Next, we examined the possibility that our OrR fly stock (OrR^YE^) had developed an immune deficiency during many years of maintenance. Two wild-type lines were obtained from Bloomington stock center, including a new OrR line (OrR^BSC^) and a CantonS line. We infected flies from these lines with wild-type *C. albicans* and found that OrR^BSC^ showed similar sensitivity to *C. albicans* infection as OrR^YE^ (p-value, day 3 = 0.263) (Figure 1D). By contrast, the CantonS flies were more sensitive to injection of PBS (p-value, day 3<0.001) and infection with *C. albicans* (p-value, day 3 = 0.001) than either OrR line. These latter results confirm that CantonS flies are less tolerant to extracellular pathogens than OrR flies [40]. Based on these findings the OrR^YE^ flies were deemed suitable and used for virulence tests.

As a final control, we infected OrR^YE^ flies with three *C. albicans* strains, *cph1Δ efg1Δ* [41], *csh3Δ* [13], and *sap2Δ* [42], that had previously been reported to exhibit attenuated virulence in mice. Consistent with the results obtained using mice, in comparison to wild-type, *cph1Δ efg1Δ* (p-value, day 3 = 0.008), *csh3Δ* (p-value, day 3 = 0.039) and *sap2Δ* (p-value, day 3 = 0.05) mutants showed attenuated virulence in flies (Figure 1E). Together, these results indicate that wild-type *Drosophila* can be used as a model of *C. albicans* virulence.

### *C. albicans* disseminates and exhibits several morphological forms after injection into the *Drosophila* thorax

**Figure 2:**
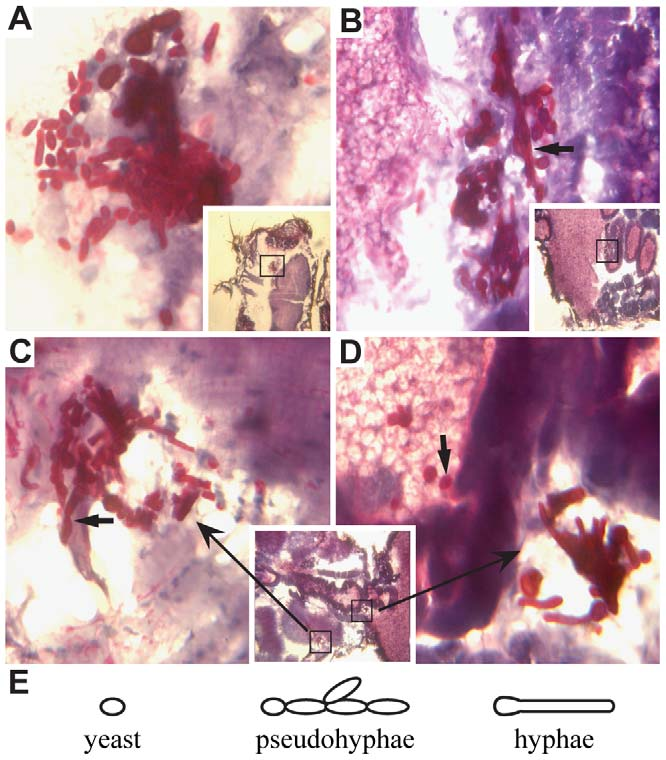
Wild-type *C. albicans* cells invade and colonize numerous sites and display multiple morphologies. Flies were injected with *C. albicans* (PMRCA18) and three days post-infection, histological sections of infected flies were prepared. A. Infection in the head. B. Infection in the abdomen. C and D. Infection at several sites within the thorax, including the muscles (C) and gut (D). The large photographs in each panel are higher magnification views of the insets as indicated. Yeast-like (thick arrow; panel D), pseudohyphal (thick arrow; panel C), and hyphal cells (thick arrow; panel B) are observed in all tissues. E. Schematic depiction of yeast-like, pseudohyphal, and hyphal morphological forms of *C. albicans*.

The course of infection was followed for seven days. Histological sections of *Drosophila* tissues were prepared and Periodic Acid-Schiff staining was carried out to allow visualization of fungal cells. Following injection into the thorax, wild-type *C. albicans* was able to disseminate and colonize multiple sites throughout the flies (Figure 2). The three morphological forms of *C. albicans*, (yeast-like round cells, psuedohyphae and hyphae (Figure 2E)), were observed in *Drosophila* tissues as early as one day post-infection, and all three forms persisted throughout the course of the infection. We detected fungal cells in the head (Figure 2A), in the abdomen (Figure 2B), and within the thorax where fungal cells were found multiply dispersed (Figure 2C and 2D, inset). These sites included muscle tissue (expanded in C), gut tissue, including yeast-like single cells that were observed in the ventriculus (expanded in D) and hyphae that appeared to be invading the ventriculus from outside the gut tissue (one of which is expanded in D). *C. albicans* was present as single cells (arrow in D), pseudohyphae (arrow in C) and hyphae (arrow in B). No obvious prevalence of any single morphological form was apparent during the seven day infection period.

### *Drosophila* mbn-2 cells phagocytose *C. albicans* better than *S. cerevisiae*

**Figure 3:**
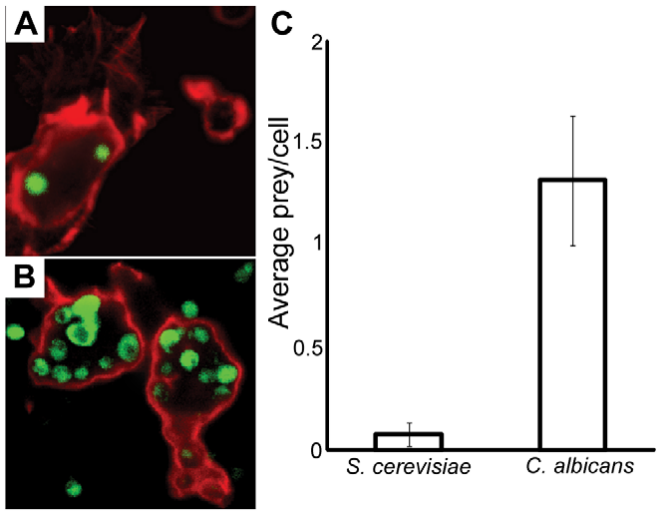
*Drosophila* mbn-2 cells phagocytose *C. albicans* more effectively than *S. cerevisiae*. Phagocytic prey, FITC- labeled *S. cerevisiae* (A) or *C. albicans* (B) are shown in green and F-actin in mbn-2 cells is shown in red. C. The average number of internalized fungal cells per mbn-2 cell (n>400) from three independent experiments was quantified.

We compared the capacity of *D. melanogaster* mbn-2 cells [43], a hemocyte-derived cell line, to phagocytose *C. albicans* and *S. cerevisiae* cells. We found that mbn-2 cells internalized 17-fold more *C. albicans* than *S. cerevisiae* per cell (1.32 *C. albicans* cells vs. 0.077 *S. cerevisiae* cells per mbn-2 cell) (Figure 3). The number of attached but not internalized *S. cerevisiae* cells was also lower. It is likely that the higher ingestion of *C. albicans* was due to increased binding to the hemocyte surface. Since the two yeast strains are similar in cell size, we assume that the clear difference in internalization efficiency was not a consequence of membrane depletion, a potential rate-limiting step of phagocytosis. Furthermore, it has been shown that *Drosophila* secrete Macroglobulin complement related (Mcr), a protein that binds specifically to *C. albicans* to promote phagocytosis [44]. Despite efficient phagocytosis of *C. albicans* cells, the pathogenic properties of this fungus must enable it to evade the fly immune response to cause invasive and lethal infections. Consistent with this notion, phagosome-induced hyphal growth has been shown to enable *C. albicans* cells to escape human macrophages following phagocytosis, a characteristic lacking in the avirulent yeast *S. cerevisiae* [45].

### *STP1* is required for virulence

**Figure 4:**
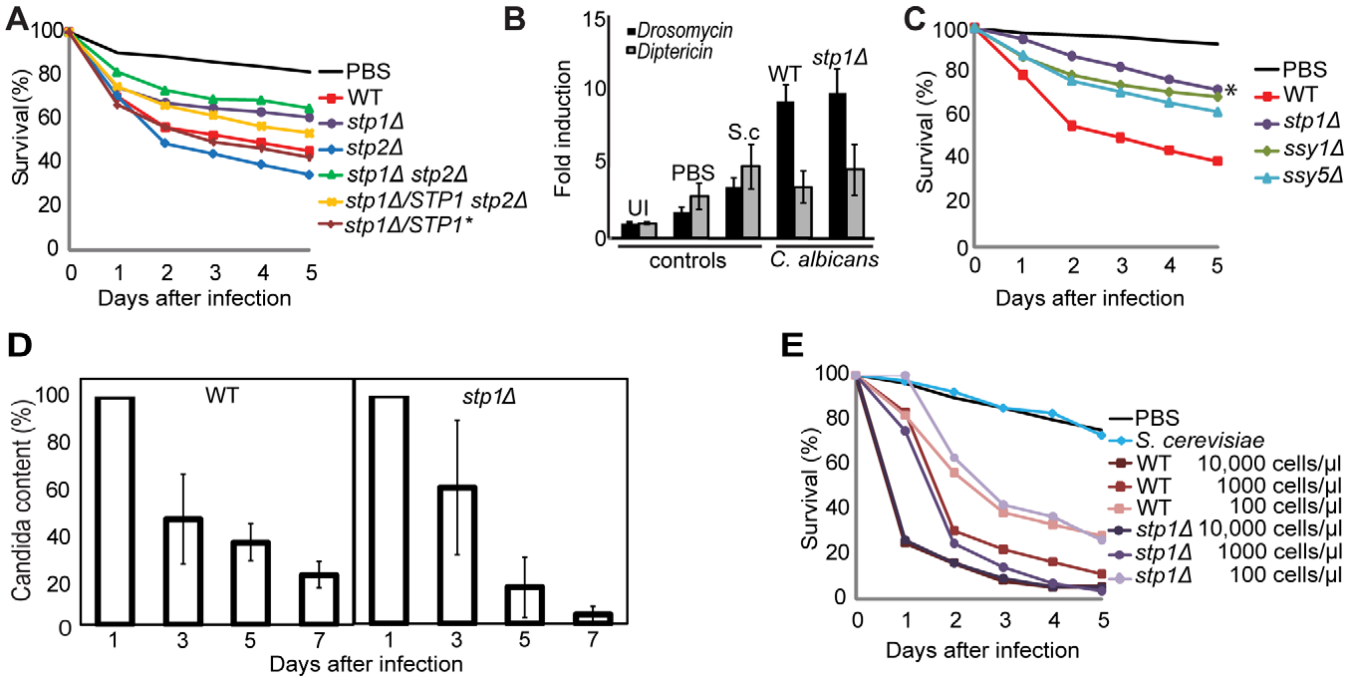
A functioning SPS-sensing pathway is required for virulence. A. OrR flies (n = 500) were injected with PBS or wild-type (WT; PMRCA18), *stp1Δ* (PMRCA59), *stp2Δ* (PMRCA57), *stp1Δstp2Δ* (PMRCA94), *stp1Δ/STP1stp2Δ* (PMRCA95), and stp1Δ/STP1* (PMRCA60) *C. albicans* strains (10,000 cells/µl). B. The levels of *Drosomycin* and *Diptericin* expression (normalized to *RpL32*) were analyzed by quantitative RT-PCR in un-injected (UI) flies and in flies 20 hours post-injection with PBS, *S. cerevisiae* (S.c.;KRY001), wild-type (WT; PMRCA18) or *stp1Δ* (PMRCA59) *C. albicans*. C. OrR flies (n = 500) were injected with PBS or wild-type (WT; PMRCA18), *stp1Δ* (PMRCA59), *ssy1Δ* (YJA64), and *ssy5Δ* (YJA53) *C. albicans* strains (10,000 cells/µl). Cohorts of 500 flies were injected. D. Pathogen loads were monitored by quantitative PCR using DNA isolated from OrR flies infected with wild-type (WT; PMRCA18) or *stp1Δ* (PMRCA59) *C. albicans*. Levels of *CaACT1* DNA were normalized to levels of *DmRpL32* DNA and relative to values determined at Day 1 post-infection (set to 100) are plotted. Error bars represent SEM. E. *Tl^632^/Tl^(1-RXA)^* flies (n≥50) were injected with PBS, *S. cerevisiae* (KRY001) at 10,000 cells/µl, or *C. albicans* strains wild-type (WT; PMRCA18) or *stp1Δ* (PMRCA59) at 10,000, 1000, or 100 cells/µl. Statistically significant differences from the survival of the flies injected with WT *C. albicans* are marked by an asterisk.

Next, we examined the virulence properties of *C. albicans* mutants lacking components of the SPS signaling pathway (Figure 1A). Deletion of *STP1* (*stp1Δ*) alone reduced the virulence of *C. albicans* [Figure 4A (p-value, day 3 = 0.115) and Figure 4C (p-value, day 3 = <0.001)]. The difference in statistical significance between these experiments, from showing a clear trend to a highly significant result, reflects the improvements made to the infection procedure during the course of this investigation (detailed in Materials and Methods S1).By contrast, deletion of *STP2 (stp2Δ)* did not reduce virulence [Figure 4A (p-value, day 3 = 0.525)]. The deletion of both *STP1* and *STP2* (*stp1Δ stp2Δ*) resulted in a similar level of lethality as the *stp1Δ* mutant, indicating that Stp1 and not Stp2 is a virulence factor. Consistent with the role of Stp1 in virulence, the re-introduction of a wild-type copy of *STP1* into the *stp1Δ stp2Δ* double deletion mutant (*stp1Δ/STP1 stp2Δ*) partially restored virulence, although this is only visible four and five days after infection. The introduction of a constitutively active STP1* allele, which encodes a truncated Stp1, into the *stp1Δ* mutant (stp1Δ/STP1*) restored virulence completely.

### Ssy1 and Ssy5 are required for *C. albicans* virulence

To further evaluate the role of SPS sensing pathway signaling via Stp1, we injected *C. albicans* mutants lacking the amino acid receptor Ssy1 (*ssy1Δ*) or the Stp1 activating endoprotease Ssy5 (*ssy5Δ*). In comparison to wild-type, both mutant strains exhibited impaired virulence and survival curves clearly match that of flies infected with the *stp1Δ* mutant(Figure 4C) (p-values, day 3 = *ssy1Δ* = 0.072 and *ssy5Δ* = 0.137). While these data are not statistically significant, the combination of double-blind studies and the verification of our system using less virulent *C. albicans* strains (Figure 1E) lends to the strength of these observations. These results, coupled with the previous findings regarding the attenuated virulence of *csh3Δ* and *sap2Δ* mutants in mice [13], [26] and *Drosophila* (Figure 1E), are fully consistent with the known hierarchy of components of the SPS sensing pathway (Figure 1A). These results imply that the SPS sensing pathway and the ability to sense amino acids present in infected hosts is important for inducing virulent growth of *C. albicans.*

### *Drosomycin* expression is induced normally following *C. albicans* infection

Fungal infections in *Drosophila* lead to the activation of the Toll pathway [33], [46], [47]. Intracellular signal transduction results in the activation of the transcription factors Dif and Dorsal [48], [49], [50], [51], [52]. Translocation of these transcription factors into the nucleus results in the activation of Toll responsive immune genes, including the gene encoding the anti-fungal peptide Drosomycin. The levels of *Drosomycin* expression were monitored to examine whether the observed differences in lethality of flies infected with the various *C. albicans* strains could be traced to effects on the *Drosophila* immune response. PBS injection alone caused a small induction of *Drosomycin* expression and infection with *S. cerevisiae* caused a 3-fold induction of expression (Figure 4B). Infection with either wild-type or *stp1Δ C. albicans* resulted in an equivalent, approximately 9-fold, induction of *Drosomycin*. Conversely, the Imd-pathway response gene, *Diptericin*, is not induced by Candida infection; rather, the small induction (Figure 4B), observed following injection can be accounted for by a minimal would response induction of AMP expression, since PBS injection alone stimulates the same level of expression as fungal infection. Thus, the difference in virulence between the two *C. albicans* strains was not due to alterations in the immune response in the fly hosts.

### Pathogen loads decrease over time in flies surviving infection

**Figure 5:**
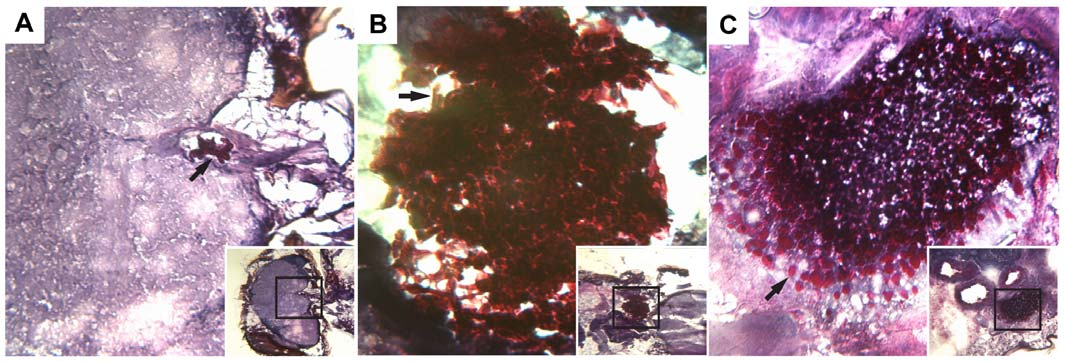
Histological evaluation of flies infected by *stp1Δ C. albicans*. Despite exhibiting attenuated virulence, *stp1Δ* mutant cells colonize many sites and exhibit diverse morphological forms. Flies were injected with *C. albicans* (PMRCA59) and three days post-infection, histological sections of infected flies were prepared. A. Infection in the head. B. Infection in the thorax. C. Infection in the abdomen. The large photographs in each panel are higher magnification views of the insets as indicated. Yeast-like (arrow; panel C), pseudohyphal (arrow; panel A), and hyphal cells (arrow; panel B) are observed.

Differences in virulence characteristics of pathogens can be a function of the critical threshold in the number of cells required for lethality [53]. To determine whether the variations in virulence could be explained by differences in pathogen loads, flies were infected with equal numbers of wild-type and *stp1Δ C. albicans* cells and pathogen loads were analyzed. DNA was isolated from living flies at 1, 3, 5 and 7 days post-infection and the levels of *CaACT1* DNA were quantified using qPCR and the values were normalized to the levels of *Drosophila RpL32* DNA. On day 1 post-infection, despite injection of equal numbers of cells of each strain, the amount of *C. albicans* DNA recovered from flies infected with wild-type cells was significantly lower than that recovered from flies infected with *stp1Δ* cells (Figure S1). Observations from histological analysis of infected flies may partially explain why larger amounts of *stp1Δ* DNA were isolated. We have noted that although *stp1Δ* cells colonize many different tissues and are present in all morphological forms (Figure 5), in comparison to wild-type (Figure 2), they exhibit less invasive growth into *Drosophila* tissues, and are more often associated with tissues bathed in hemolymph. Consequently, the extraction of *stp1Δ* cells from *Drosophila* tissues may simply be more efficient. Despite this initial difference in levels of fungal DNA, the relative amount of both wild-type and *stp1Δ C. albicans* DNA dropped over the course of the infection (Figure 4D). These findings suggest that surviving flies are able to reduce and maintain numbers of fungal cells below a critical lethal threshold. We have been able to isolate viable *C. albicans* cells from surviving flies up to seven days post-infection. Thus, despite decreasing pathogen loads, the flies do not completely clear the infection.

### Toll pathway mutants fail to reveal differences in virulence

All previously published studies examining *Candida* virulence in *Drosophila* have employed flies defective in Toll signaling [36], [37], [38]. We tested whether we could use *Tl* mutants to assess the difference in virulence between *stp1Δ* and wild-type *C. albicans*. *Tl^632^/Tl^(1-RXA)^* flies were infected with *S. cerevisiae* (10,000 cells/µl), wild-type or *stp1Δ C. albicans* (at 10,000, 1000, and 100 cells/µl) (Figure 4E). *S. cerevisiae* is completely avirulent to the *Tl* mutants, whereas both wild-type and *stp1Δ C. albicans* exhibited robust virulence. A difference in virulence between *stp1Δ* and wild-type *C. albicans* was not observed following injection of 10,000, 1000, or 100 cells/µl, despite obvious concentration-based changes in survival following infection with each strain. The high rates of lethality, independent of the fungal genotype, must reflect the poor immune defense of the *Tl* mutant host, and clearly indicate that *Tl* mutant flies are not suitable for a nuanced analysis of fungal virulence traits.

## Discussion

Here we report that *C. albicans* virulence can be assessed in wild-type *Drosophila*. In an unbiased manner, using a double-blind strategy, this mini-host model clearly detected the well-documented reduced virulence of *cph1Δ efg1Δ*, *csh3Δ,* and *sap2Δ C. albicans* strains, and showed that a prototrophic diploid *S. cerevisiae* strain is avirulent (Figure 1E). Prior to this study, the assessment of *C. albicans* virulence in *Drosophila* was thought to require the use of severely immuno-compromised Toll pathway mutants [36], [37], [38]. In striking contrast we found that the hypersensitivity of Toll pathway mutant flies significantly restricted the dynamic range of the virulence assay (Figure 4E); wild-type OrR flies survived infections with mutant *C. albicans* strains that induce significant lethality in *Tl* mutant flies (compare Figures 1, 4A and 4C to Figure 4E). The use of wild-type *Drosophila* provides a more robust and nuanced assessment of fungal virulence. An additional strength of the assay described here is that it eliminates the need to perform crosses to generate homozygous *Tl* mutant flies. This system is amenable to any fungal biologist without requiring an in depth knowledge of *Drosophila* genetics and manipulation.

*C. albicans*-induced lethality occurs in two stages (Figures 1 and 4). The first stage, from 1–3 days after infection, can be classified as an acute infection. During this period, the injected *C. albicans* begin to establish sites of infection throughout the host and invades multiple host tissues (Figures 2 and 5). During this acute phase, the *Drosophila* immune system is activated in an attempt to combat the infection (Figure 4B). Thus, the dynamic interaction between the host's immune response and the pathogen's ability to invade and establish an infection in tissues dictates the lethality observed in the first three days. After day 3 post infection the slopes of the killing curves changed such that the rates of lethality were similar regardless of the genotype of the fungal cells injected. Although the pathogen load decreased over time (Figure 4D), both wild-type and *stp1Δ* fungal cells could be isolated up to 7 days post-infection, suggesting that surviving flies are not able to completely clear infections, but rather appear to tolerate the presence of *C. albicans*. Similarly, it has been shown that although *cph1Δ efg1Δ* mutant cells are essentially avirulent, they proliferate and persist asymptomatically in mice [54]. Thus, it appears that during the post-acute stage of infection, the host response is successful in shifting the balance of host-pathogen interactions in favor of host survival. Based on these observations, differences in virulence properties of fungal cells are best evaluated at three days post infection.

The SPS sensing pathway is required for the activation of multiple systems necessary for nitrogen source uptake [10]. Using the *Drosophila* host model we have found that the transcription factor, Stp1, and its upstream activators, Ssy1 and Ssy5, are required for full virulence (Figure 4A and 4C). The reduced lethality of *csh3Δ*, carrying a deletion in an ER membrane-localized chaperone required for proper functioning of the SPS sensing pathway [13], and *sap2Δ* (Figure 1E), carrying a deletion of the gene encoding a major secreted protease that is strictly controlled by Stp1 [10], [23], is consistent with our finding that SPS-sensing pathway signaling through Stp1 is important for virulence (Figure 4A and 4C).

The resilience of flies infected with *stp1Δ C. albicans* is likely the consequence of multiple factors. Perhaps most important is that *stp1Δ* mutants do not express *SAP2* [10]. The inability of *stp1Δ* mutants to express and secrete Sap2 reduces the likelihood of tissue damage in the host, and may compromise the ability of mutants to grow invasively (Figure 5) [55], [56]. Also, in addition to causing tissue damage, the induced expression of Sap2 may lead to the degradation of extracellular signaling components important for energy homeostasis or the host immune response like secreted antimicrobial peptides (AMPs). In fact, it has been shown that *Drosophila* genes involved in protein translation, energy homeostasis, and stress responses are important for the host to survive an infection [57], [58], [59]. Thus, while it is likely that less tissue damage caused by infection is the primary reason flies survive infection with *stp1Δ* compared to wild type *C. albicans*, it is possible that *stp1Δ* mutants may fail to interfere with the other facets of the host's ability to survive infection. Although more work is needed to differentiate between these possibilities, our results are consistent with the documented importance of Sap2 in mammalian model host systems and humans. For example, mice immunized with purified Sap2 have greatly reduced loads of *C. albicans* during systemic infections [26], and also in oral and vaginal infections [25], [27]. Furthermore, *C. albicans* isolates obtained from immune-compromised human hosts express higher levels of SAP activity than those obtained from control patients [24].

Finally, we note that the significance of Stp1 in virulence could not have been anticipated. *STP1*, but not *STP2*, is transcriptionally repressed in the presence of millimolar concentrations of extracellular amino acids [23]. Consequently, the high concentrations of free amino acids (0.2 – 20 mM) circulating in the *Drosophila* hemolymph [60] were expected to suppress the expression of *STP1*, and limit the expression of *SAP2*. The finding that Stp1 contributes to virulence suggests that within flies, a critical number of *C. albicans* cells experience nitrogen source limitation, enabling the SPS-sensor to activate Stp1-induced virulence traits. These unanticipated results underscore the importance of assessing the virulence properties of single fungal genes *in vivo* using model host systems.

## Materials and Methods

### Drosophila stocks

All *Drosophila* stocks were maintained on standard cornmeal agar medium at 25°C. The primary wild-type *Drosophila* stock was an OrR (OrR^YE^) strain originally obtained from Bloomington stock center and maintained in the Engström lab for many years. The additional OrR (OrR^BSC^) (stock #5) and CantonS (stock #1) lines were newly obtained specifically for this study from Bloomington stock center. *Tl* mutant flies were obtained by crossing the temperature sensitive allele bearing, *w*; *Tl ^632^ ca*/*TM6B*, *Tb*) females to the null mutant carrying, *Tl^(1-RXA)^ e*/*TM6B, Hu e* males at 18°C. *Tl^632^/Tl^(1-RXA)^* adults were collected and transferred to 29°C for three days prior to injection of fungal cell suspensions.

### Fungal strains

The genotypes of the strains used in this study are listed in Table S2. Standard methods as described in [61] were used to construct CAI4 derivative strains carrying *ssy5* and *ssy1* deletions. Briefly, base pairs +78 to +2,483 of both *SSY5* ORFs in strain PMRCA18 were replaced by the *SAT1* flipper cassette from pSFS2; two rounds of integration/excision generated the homozygous *ssy5* deletion strain YJA53. Similarly, base pairs +37 to +2,808 of both *SSY1* ORFs in strain PMRCA18 were replaced to generate the homozygous *ssy1* deletion strain YJA64. Deletions were confirmed by PCR and by phenotypic growth-based assays on selective media. Strains carrying deletions that abrogate SPS sensor signaling are resistant to the toxic lysine analogue 2-aminoethyl-L-cysteine (225 µg/ml) [13] and sensitive to the sulfonylurea herbicide MM (2-{[({[(4-methoxy-6-methyl)-1,3,5-triazin-2-yl]-amino}carbonyl) amino]-sulfonyl}-benzoic acid); at 1.5 mg/ml [10]. Haploid S288c based strains of *S. cerevisiae* are often used as negative controls in fungal virulence assays. Here we have constructed and used a diploid *S. cerevisiae* strain derived from the Σ1278b background. Although Σ1278b and its derivatives cross well with other standard laboratory strains such as S288C [62], Σ1278b background strains undergo the most uniform and easily controlled transition from unpolarized to filamentous pseudohyphal growth.

### Infection of flies

Fungal strains were grown at 30°C in liquid yeast extract-peptone-dextrose (YPD) medium, prepared as described [63]. Cells were harvested in early logarithmic-phase of growth (OD_600_ ≈ 1), washed once in phosphate-buffered saline (PBS; pH 7), and re-suspended in PBS. Heat-killed *C. albicans* was produced by incubating the cell suspension at 100°C for one hour. Flies were injected using a fine glass capillary needle with a micro-injector (TriTech Research, Los Angeles, CA, USA). A minimum of 500 wild-type flies were injected with an approximate volume of 50 nl of suspension using a minimum of four independently prepared fungal preparations. The injection of 500 flies takes less than an hour, and thus is quite amenable to high-throughput analyses. A minimum of 50 *Tl^632^/Tl^(1-RXA)^* adults were infected with each fungal suspension. The difference in number of flies is a reflection of the difficulty in obtaining large numbers of *Tl^632^/Tl^(1-RXA)^* adults. Flies were maintained at 29°C for up to seven days after infection, and transferred to new vials on the 3^rd^ day after infection. Although it may not be ideal to assess virulence of human pathogens at temperatures below 37°C, incubation of flies at 29°C is a necessary compromise since this temperature is the upper limit for the long term survival of flies. Our initial series of infections showed a relatively high variation and the statistical significance was not satisfying. The methodology was improved by following strictly identical schemes for growth of fungal cultures, for rearing, aging and collection of flies, and most importantly, by infecting cohorts of *Drosophila* with wild-type and mutant *C. albicans* strains in parallel (See Materials and Methods S1 for a detailed description of the *Drosophila* breading and *C. albicans* culturing protocols).

Statistical comparisons, three days following infection, were carried out using a mixed logistics model: A binomial regression model was fitted to the average values and odds ratios were estimated. Standard errors were scaled using square root of deviance-based dispersion. Stata version 11 was used for the analysis. Raw data for all points are shown in Table S1.

### RNA isolation and qPCR

For the qPCR experiments examining *Drosomycin* and *Diptericin* gene induction, RNA was isolated using TRIzol (Invitrogen, Carlsbad, CA, USA) and was treated with Turbo-DNase (Ambion, Foster City, CA, USA) according to manufacturer's instructions. The isolated RNA (diluted to 100 ng/µl, 5 µl was then used for a 25 µl reaction) was used for cDNA synthesis with random hexamers using TaqMan Reverse Transcription Reagents (Applied Biosystems, Foster City, CA, USA). Primer sequences used: *Drs* (CG10810) (drs-F: 5′-gtgagaaccttttccaatatgatgca-3′; drs-R: 5′-cggcatcggcctcgtt-3′; probe: 5′-ccaggaccaccagcatc-3′); *Dpt* (CG12763) (Dpt-F: 5′-gcaatcgcttctactttggcttat-3′; Dpt-R: 5′-gtggagtgggcttcatggt-3′; probe: 5′-ccgatgcccgacgacat-3′)*RpL32* (CG7939) (RpL32-F: 5′-caccagtcggatcgatatgct-3′; RpL32-R: 5′-acgcactctgttgtcgatacc-3′; probe: 5′-catttgtgcgacagctt-3′). TaqMan probes were used to analyze gene expression levels. The PCR program was 95°C for 10 minutes, followed by 40 cycles of 95°C for 10 seconds, 60°C for 45 seconds in a RotoGene Q machine (Qiagen, QIAGEN Strasse 1, Hilden, Germany). The efficiencies of the *Drosomycin*, *Diptericin*, and *RpL32* PCR reactions were 1.72, 1.83 and 1.75, respectively. All samples were analyzed in triplicate, and the measured mRNA concentration was normalized relative to the control *RpL32* values. The normalized data were used to quantify the relative levels of mRNA according to the relative expression ratio mathematical model [64].

### DNA isolation and qPCR

Pools of flies were collected at indicated time points and homogenized in TENTS (100 mM NaCl, 10 mM Tris, 1 mM EDTA, 2% Triton, 1% SDS), extracted with phenol:chloroform:isoamyl alcohol (25:24:1), then chloroform, precipitated with sodium acetate/ethanol, and re-suspended in sterile water. Isolated DNA was diluted to a 50 ng/µl working solution, and 250 ng was used for qPCR using a KAPA SYBR Fast qPCR kit (KAPA Biosystems, Woburn, MA, USA) according to manufacturer's instructions. The primers Act1-F (5′- gtt gac cga agc tcc aat gaa tcc -3′) and Act1-R (5′- ggt caa tac cag cag ctt cca aac c -3′) were used to detect the *C. albicans* Actin gene and RpL32-F2 (5′- agc ata cag gcc caa gat cg -3′) and RpL32-R2 (5′- agt aaa cgc ggg ttc tgc at -3′) were used to detect the *Drosophila RpL32* gene. The PCR program was 95°C for 3 minutes, followed by 40 cycles of 95°C for 3 seconds, 60°C for 20 seconds, and 72°C for 3 seconds on a RotoGene Q machine (Qiagen, QIAGEN Strasse 1, Hilden, Germany). The efficiencies of the *CaACT1* and *DmRpL32* PCR reactions were 1.71 and 1.74, respectively. At least three independent experiments were performed and results were analyzed using the relative expression ratio mathematical model [64].

### Phagocytosis assays

*Drosophila* mbn-2 cells [43] were plated on sterile glass cover slips in 4-well plates at a density of 5×10^5^ cells/ml in complete S2-cell culture medium (Invitrogen, Carlsbad, CA, USA) and grown for 48 hours. The insect steroid hormone 20-hydroxyecdysone [65] was added to the growth medium to a final concentration of 1 µM, 24 hours prior to treatment with fungal cells to induce differentiation and improve the phagocytic capacity of the *Drosophila* cell cultures [66]. Phagocytic prey, (FITC (5 mg/ml) labeled *S. cerevisiae* (KRY001) or *C. albicans* (PMRCA18), was added at a multiplicity of infection of five prey cells per mbn-2 cell and incubated for 4 hours at 25°C, washed three times with PBS to remove external yeasts, and followed by fixation in 2% (w/v) paraformaldehyde (Sigma, St. Louis, MO, USA). Preparations were blocked in PBS containing 2% bovine serum albumin (BSA), and F-actin was labeled by incubation for 30 minutes with Alexa594 Fluor-phallacidin (Molecular Probes Inc, Eugene, OR, USA) in PBS containing 100 µg/ml lysophosphatidylcholin (Sigma) as a membrane permeabilizing agent. Preparations were mounted in ProLong mounting media (Molecular Probes Inc, Eugene, OR, USA). Anaglyph confocal images were acquired with an LSM 510 Laser Scanning Microscope (Zeiss, Oberkochen, Germany) and phagocytosis was manually quantified from the images of cells (n>400). Phagocytic index was calculated as the average number of internalized fungal cells per mbn-2 cell and then normalized such that the value for *S. cerevisiae* equaled one. At least three separate experiments were performed.

### Histological sections and microscopy

Flies were injected with *C. albicans* and maintained at 29°C for the desired time. Flies were embedded in O.C.T. compound (Miles, USA) and flash frozen in liquid nitrogen. Embedded flies were equilibrated to −20°C for 24 hours prior to sectioning. Twenty µm sections were obtained using a Leica CM1850 Cryostat (Wetzler, Germany) and mounted on Chromalun [KCr(SO_4_)_2_ ×12H_2_O] (0.07%)/gelatin (2%) coated slides and dried overnight at room temperature. Sections were fixed in 3.7% formaldehyde and stained using Periodic Acid-Schiff staining (Sigma, St. Louis, MO, USA) according to manufacturer's instructions.

## Supporting Information

Figure S1 **Pathogen loads (** ***CaACT1*** **DNA) were monitored by quantitative PCR using DNA isolated from OrR flies infected with wild-type (WT; PMRCA18) or** ***stp1Δ*** **(PMRCA59)** ***C. albicans*** **at 10,000 cells/µl.** A. Flies injected with *stp1Δ C. albicans* have higher pathogen loads. B. Raw values of relative amounts of *CaACT1* DNA isolated from OrR flies infected with wild-type (WT; PMRCA18) or *stp1Δ* (PMRCA59) *C. albicans* at 10,000 cells/µl. Levels of *CaACT1* DNA (normalized to levels of *DmRpL32* DNA) are shown, values are relative to levels of wild-type (PMRCA18) *CaACT1* at1 day post-injection (set at 1).
(TIF)

Materials and Methods S1 **A detailed description of the** ***Drosophila*** **breading and** ***C. albicans*** **culturing protocols.**
(DOC)

Table S1 **Percent average survival for all infections shown in this study.**
(DOC)

Table S2 **Fungal strains used in this study.**
(DOC)

Table S3 **Pair-wise statistical analysis of survival curves at day 3 post-infection.**
(DOC)

## References

[1] Sallah S, Wan JY, Nguyen NP, Vos P, Sigounas G (2001). Analysis of factors related to the occurrence of chronic disseminated candidiasis in patients with acute leukemia in a non-bone marrow transplant setting: A follow-up study.. Cancer.

[2] Hermann P, Berek Z, Nagy G, Kamotsay K, Rozgonyi F (2001). Pathogenesis, microbiological and clinical aspects of oral candidiasis (candidosis).. Acta Microbiol Immunol Hung.

[3] Miceli MH, Diaz JA, Lee SA (2011). Emerging opportunistic yeast infections.. Lancet Infect Dis.

[4] Kibbler CC, Seaton S, Barnes RA, Gransden WR, Holliman RE (2003). Management and outcome of bloodstream infections due to *Candida* species in England and Wales.. J Hosp Infect.

[5] Macphail GL, Taylor GD, Buchanan-Chell M, Ross C, Wilson S (2002). Epidemiology, treatment and outcome of candidemia: A five-year review at three Canadian hospitals.. Mycoses.

[6] Weinstein MP, Towns ML, Quartey SM, Mirrett S, Reimer LG (1997). The clinical significance of positive blood cultures in the 1990s: A prospective comprehensive evaluation of the microbiology, epidemiology, and outcome of bacteremia and fungemia in adults.. Clin Infect Dis.

[7] Sanglard D (2002). Resistance of human fungal pathogens to antifungal drugs.. Curr Opin Microbiol.

[8] Soll DR (2002). *Candida* commensalism and virulence: The evolution of phenotypic plasticity.. Acta Trop.

[9] Rozell B, Ljungdahl PO, Martinez P (2006). Host-pathogen interactions and the pathological consequences of acute systemic *Candida albicans* infections in mice.. Curr Drug Targets.

[10] Martinez P, Ljungdahl PO (2005). Divergence of Stp1 and Stp2 transcription factors in *Candida albicans* places virulence factors required for proper nutrient acquisition under amino acid control.. Mol Cell Biol.

[11] Ljungdahl PO (2009). Amino-acid-induced signalling via the SPS-sensing pathway in yeast.. Biochem Soc Trans.

[12] Forsberg H, Ljungdahl PO (2001). Genetic and biochemical analysis of the yeast plasma membrane Ssy1p-Ptr3p-Ssy5p sensor of extracellular amino acids.. Mol Cell Biol.

[13] Martinez P, Ljungdahl PO (2004). An ER packaging chaperone determines the amino acid uptake capacity and virulence of *Candida albicans*.. Mol Microbiol.

[14] Brega E, Zufferey R, Mamoun CB (2004). *Candida albicans* Csy1p is a nutrient sensor important for activation of amino acid uptake and hyphal morphogenesis.. Eukaryot Cell.

[15] Wu B, Ottow K, Poulsen P, Gaber RF, Albers E (2006). Competitive intra- and extracellular nutrient sensing by the transporter homologue Ssy1p.. J Cell Biol.

[16] Abdel-Sater F, Jean C, Merhi A, Vissers S, André B (2011). Amino-acid signalling in yeast: activation of the Ssy5 protease is associated with its phosphorylation-induced ubiquitylation.. J Biol Chem.

[17] Omnus DJ, Pfirrmann T, Andréasson C, Ljungdahl PO (2011). A Phosphodegron Controls Nutrient-Induced Proteasomal Activation of the Signaling Protease Ssy5.. Molecular biology of the cell.

[18] Abdel-Sater F, El Bakkoury M, Urrestarazu A, Vissers S, André B (2004). Amino acid signaling in yeast: casein kinase I and the Ssy5 endoprotease are key determinants of endoproteolytic activation of the membrane-bound Stp1 transcription factor.. Mol Cell Biol.

[19] Andréasson C, Heessen S, Ljungdahl PO (2006). Regulation of transcription factor latency by receptor-activated proteolysis.. Genes Dev.

[20] Pfirrmann T, Heessen S, Omnus DJ, Andréasson C, Ljungdahl PO (2010). The prodomain of Ssy5 protease controls receptor-activated proteolysis of transcription factor Stp1.. Mol Cell Biol.

[21] Andreasson C, Ljungdahl PO (2002). Receptor-mediated endoproteolytic activation of two transcription factors in yeast.. Genes Dev.

[22] Boban M, Ljungdahl PO (2007). Dal81 enhances Stp1- and Stp2-dependent transcription necessitating negative modulation by inner nuclear membrane protein Asi1 in *Saccharomyces cerevisiae*.. Genetics.

[23] Dabas N, Morschhauser J (2008). A transcription factor regulatory cascade controls secreted aspartic protease expression in *Candida albicans*.. Mol Microbiol.

[24] Korting HC, Schaller M, Eder G, Hamm G, Bohmer U (1999). Effects of the human immunodeficiency virus (HIV) proteinase inhibitors saquinavir and indinavir on in vitro activities of secreted aspartyl proteinases of *Candida albicans* isolates from HIV-infected patients.. Antimicrob Agents Chemother.

[25] De Bernardis F, Boccanera M, Adriani D, Girolamo A, Cassone A (2002). Intravaginal and intranasal immunizations are equally effective in inducing vaginal antibodies and conferring protection against vaginal candidiasis.. Infect Immun.

[26] Vilanova M, Teixeira L, Caramalho I, Torrado E, Marques A (2004). Protection against systemic candidiasis in mice immunized with secreted aspartic proteinase 2.. Immunology.

[27] Rahman D, Mistry M, Thavaraj S, Challacombe SJ, Naglik JR (2007). Murine model of concurrent oral and vaginal *Candida albicans* colonization to study epithelial host-pathogen interactions.. Microbes Infect.

[28] Klasson H, Fink GR, Ljungdahl PO (1999). Ssy1p and Ptr3p are plasma membrane components of a yeast system that senses extracellular amino acids.. Mol Cell Biol.

[29] Butler G, Rasmussen MD, Lin MF, Santos MA, Sakthikumar S (2009). Evolution of pathogenicity and sexual reproduction in eight *Candida* genomes.. Nature.

[30] Noble SM, French S, Kohn LA, Chen V, Johnson AD (2010). Systematic screens of a *Candida albicans* homozygous deletion library decouple morphogenetic switching and pathogenicity.. Nat Genet.

[31] Lionakis MS, Lim JK, Lee CC, Murphy PM (2011). Organ-Specific Innate Immune Responses in a Mouse Model of Invasive Candidiasis..

[32] Vodovar N, Acosta C, Lemaitre B, Boccard F (2004). *Drosophila*: A polyvalent model to decipher host-pathogen interactions.. Trends Microbiol.

[33] Lemaitre B, Nicolas E, Michaut L, Reichhart JM, Hoffmann JA (1996). The dorsoventral regulatory gene cassette *spatzle/Toll/cactus* controls the potent antifungal response in *Drosophila* adults.. Cell.

[34] Uvell H, Engstrom Y (2007). A multilayered defense against infection: combinatorial control of insect immune genes.. Trends Genet.

[35] Lemaitre B, Hoffmann J (2007). The host defense of *Drosophila melanogaster*.. Annu Rev Immunol.

[36] Alarco AM, Marcil A, Chen J, Suter B, Thomas D (2004). Immune-deficient *Drosophila melanogaster*: a model for the innate immune response to human fungal pathogens.. J Immunol.

[37] Chamilos G, Nobile CJ, Bruno VM, Lewis RE, Mitchell AP (2009). *Candida albicans* Cas5, a regulator of cell wall integrity, is required for virulence in murine and toll mutant fly models.. J Infect Dis.

[38] Chamilos G, Lionakis MS, Lewis RE, Lopez-Ribot JL, Saville SP (2006). *Drosophila melanogaster* as a facile model for large-scale studies of virulence mechanisms and antifungal drug efficacy in Candida species.. J Infect Dis.

[39] Gimeno CJ, Ljungdahl PO, Styles CA, Fink GR (1992). Unipolar cell divisions in the yeast S. cerevisiae lead to filamentous growth: regulation by starvation and *RAS*.. Cell.

[40] Okado K, Shinzawa N, Aonuma H, Nelson B, Fukumoto S (2009). Rapid recruitment of innate immunity regulates variation of intracellular pathogen resistance in *Drosophila*.. Biochem Biophys Res Commun.

[41] Lo HJ, Kohler JR, DiDomenico B, Loebenberg D, Cacciapuoti A (1997). Nonfilamentous *C. albicans* mutants are avirulent.. Cell.

[42] Staib P, Lermann U, Blass-Warmuth J, Degel B, Wurzner R (2008). Tetracycline-inducible expression of individual secreted aspartic proteases in Candida albicans allows isoenzyme-specific inhibitor screening.. Antimicrob Agents Chemother.

[43] Gateff E, Gissmann L, Shrestha R, Plus N, Pfister H, K E, M K, D A (1980). Characterization of two tumorous blood cell lines of *Drosophila melanogaster* and the viruses they contain.. Invertebrate Systems In Vitro: Elsevier/North Holland Biomedical Press.

[44] Stroschein-Stevenson SL, Foley E, O'Farrell PH, Johnson AD (2006). Identification of *Drosophila* gene products required for phagocytosis of *Candida albicans*.. PLoS Biol.

[45] Lorenz MC, Bender JA, Fink GR (2004). Transcriptional response of *Candida albicans* upon internalization by macrophages.. Eukaryot Cell.

[46] Rutschmann S, Kilinc A, Ferrandon D (2002). Cutting edge: The Toll pathway is required for resistance to gram-positive bacterial infections in *Drosophila*.. J Immunol.

[47] Rosetto M, Engstrom Y, Baldari CT, Telford JL, Hultmark D (1995). Signals from the IL-1 receptor homolog, Toll, can activate an immune response in a *Drosophila* hemocyte cell line.. Biochem Biophys Res Commun.

[48] Petersen UM, Bjorklund G, Ip YT, Engstrom Y (1995). The dorsal-related immunity factor, Dif, is a sequence-specific trans-activator of *Drosophila Cecropin* gene expression.. Embo J.

[49] Manfruelli P, Reichhart JM, Steward R, Hoffmann JA, Lemaitre B (1999). A mosaic analysis in *Drosophila* fat body cells of the control of antimicrobial peptide genes by the Rel proteins Dorsal and DIF.. Embo J.

[50] Meng X, Khanuja BS, Ip YT (1999). Toll receptor-mediated *Drosophila* immune response requires Dif, an NF-kappaB factor.. Genes Dev.

[51] Rutschmann S, Jung AC, Hetru C, Reichhart JM, Hoffmann JA (2000). The Rel protein DIF mediates the antifungal but not the antibacterial host defense in *Drosophila*.. Immunity.

[52] Ip YT, Reach M, Engstrom Y, Kadalayil L, Cai H (1993). *Dif*, a dorsal-related gene that mediates an immune response in *Drosophila*.. Cell.

[53] Schneider DS, Ayres JS (2008). Two ways to survive infection: What resistance and tolerance can teach us about treating infectious diseases.. Nat Rev Immunol.

[54] Yang YL, Wang CW, Chen CT, Wang MH, Hsiao CF (2009). Non-lethal *Candida albicans cph1/cph1 efg1/efg1* mutant partially protects mice from systemic infections by lethal wild-type cells.. Mycol Res.

[55] Morschhauser J, Virkola R, Korhonen TK, Hacker J (1997). Degradation of human subendothelial extracellular matrix by proteinase-secreting *Candida albicans*.. FEMS Microbiol Lett.

[56] Colina AR, Aumont F, Deslauriers N, Belhumeur P, de Repentigny L (1996). Evidence for degradation of gastrointestinal mucin by *Candida albicans* secretory aspartyl proteinase.. Infect Immun.

[57] Becker T, Loch G, Beyer M, Zinke I, Aschenbrenner AC (2010). FOXO-dependent regulation of innate immune homeostasis.. Nature.

[58] Levitin A, Marcil A, Tettweiler G, Laforest MJ, Oberholzer U (2007). Drosophila melanogaster Thor and response to Candida albicans infection.. Eukaryot Cell.

[59] Chen J, Xie C, Tian L, Hong L, Wu X Participation of the p38 pathway in Drosophila host defense against pathogenic bacteria and fungi.. Proc Natl Acad Sci U S A.

[60] Piyankarage SC, Augustin H, Featherstone DE, Shippy SA (2009). Hemolymph amino acid variations following behavioral and genetic changes in individual *Drosophila* larvae..

[61] Reuss O, Vik A, Kolter R, Morschhauser J (2004). The SAT1 flipper, an optimized tool for gene disruption in *Candida albicans*.. Gene.

[62] Siddiqui AH, Brandriss MC (1988). A regulatory region responsible for proline-specific induction of the yeast PUT2 gene is adjacent to its TATA box.. Mol Cell Biol.

[63] Sherman F (1991). Getting started with yeast.. Methods Enzymol.

[64] Pfaffl MW (2001). A new mathematical model for relative quantification in real-time RT-PCR.. Nucleic Acids Res.

[65] Ress C, Holtmann M, Maas U, Sofsky J, Dorn A (2000). 20-Hydroxyecdysone-induced differentiation and apoptosis in the *Drosophila* cell line, l(2)mbn.. Tissue Cell.

[66] Dimarcq JL, Imler JL, Lanot R, Ezekowitz RA, Hoffmann JA (1997). Treatment of l(2)mbn *Drosophila* tumorous blood cells with the steroid hormone ecdysone amplifies the inducibility of antimicrobial peptide gene expression.. Insect Biochem Mol Biol.

