# Supplementary figures and images for: Wild-Type Drosophila melanogaster as a Model Host to Analyze Nitrogen Source Dependent Virulence of Candida albicans
Source: PLoS One. 2011 Nov 14;6(11):e27434. doi: 10.1371/journal.pone.0027434 (PMC3215725; doi:10.1371/journal.pone.0027434)

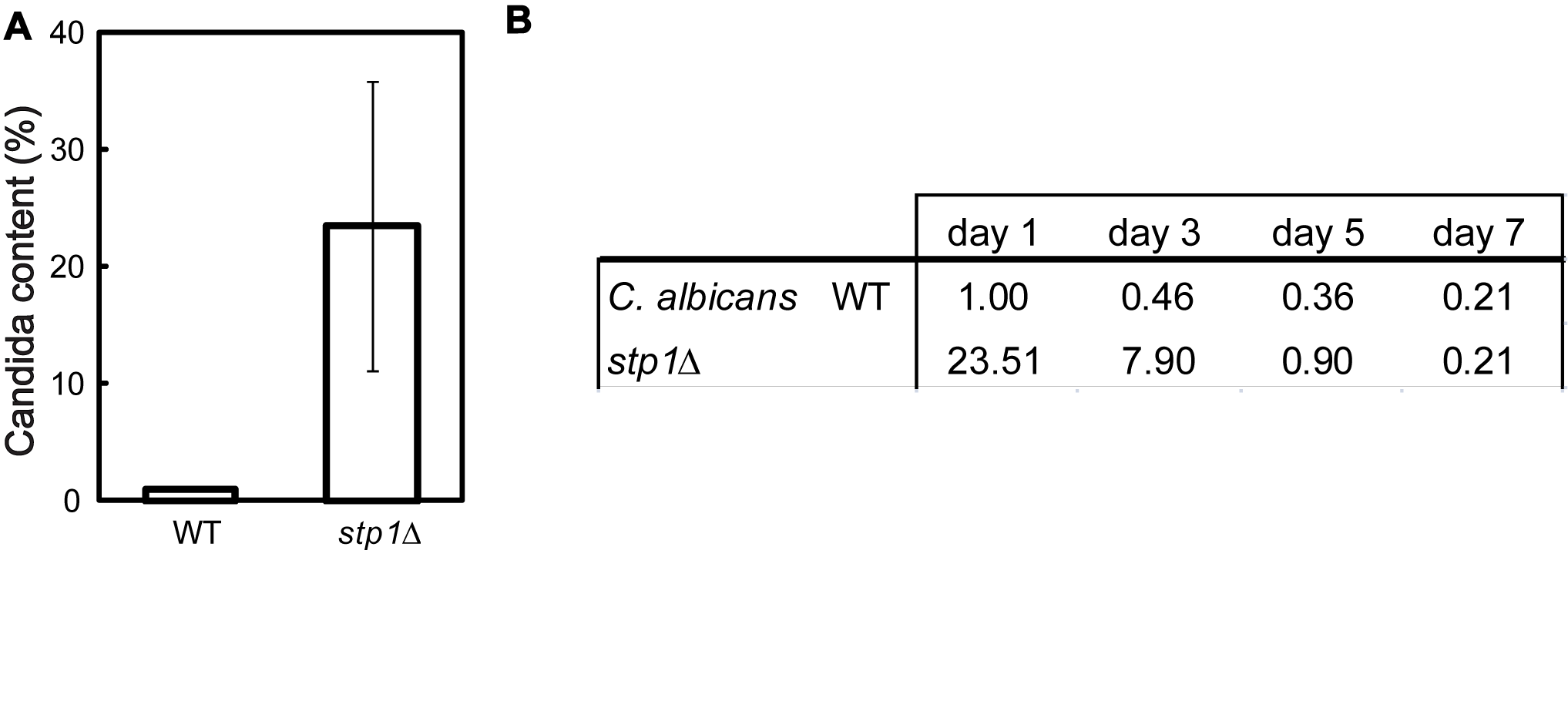

Supplement: Figure S1 — Pathogen loads ( CaACT1 DNA) were monitored by quantitative PCR using DNA isolated from OrR flies infected with wild-type (WT; PMRCA18) or stp1Δ (PMRCA59) C. albicans at 10,000 cells/µl. A. Flies injected with stp1Δ C. albicans have higher pathogen loads. B. Raw values of relative amounts of CaACT1 DNA isolated from OrR flies infected with wild-type (WT; PMRCA18) or stp1Δ (PMRCA59) C. albicans at 10,000 cells/µl. Levels of CaACT1 DNA (normalized to levels of DmRpL32 DNA) are shown, values are relative to levels of wild-type (PMRCA18) CaACT1 at1 day post-injection (set at 1). (TIF) [file pone.0027434.s001.tif]
