# Supplementary material for: Wild-Type Drosophila melanogaster as a Model Host to Analyze Nitrogen Source Dependent Virulence of Candida albicans
Source: PLoS One. 2011 Nov 14;6(11):e27434. doi: 10.1371/journal.pone.0027434 (PMC3215725; doi:10.1371/journal.pone.0027434)
Supplement: Materials and Methods S1 — A detailed description of the Drosophila breading and C. albicans culturing protocols. (DOC) [file pone.0027434.s002.doc]

**Supplemental Methods**

To ensure the reproducibility of infection conditions, rigorous routines were followed to obtain populations of OrR flies of uniform age, and *C. albicans* cultures with cells in early logarithmic phase of growth exclusively exhibiting yeast-like growth morphologies.

Fly stocks were maintained at 25 °C. Flies (approximately 300) were transferred to standard *Drosophila* culture bottles (Fisher Scientific, stock #AS117), containing standard cornmeal agar medium, after one week the flies were transferred into new bottles, and maintained for additional week to enable eggs to be laid, after which the flies were discarded. Newly eclosed progeny were transferred to fresh bottles and allowed to mature for 3-4 days prior to injection with *C. albicans* cells.

*C. albicans* strains were maintained as frozen (-80 ^o^C) stocks in 15% glycerol. Frozen scrapings were thawed on solid YPD media and streaked on plates. After 3 days, a single colony was used to inoculate 5 ml liquid YPD, and cells were allowed to grow overnight. The following morning, cultures were diluted in fresh YPD to an OD_600_ of 0.1 – 0.2 (usually 1:100) and grown shaking until an OD_600_ of 0.8 – 1.0 was reached (approximately 4 hours). In all instances the cultures were subject to continuous and vigorous shaking, and the incubation temperature was 30 ^o^C. An appropriate volume of culture to obtain a 500 µl of cell suspension with an OD_600_ of 1 was centrifuged at 16,000 x g, washed once with 1 ml 1X PBS, and resuspended in 500 µl 1X PBS.

For proper statistical analysis, cohorts of flies should be injected with wild-type cells in parallel with the desired mutant cells.
