# Supplementary material for: Wild-Type Drosophila melanogaster as a Model Host to Analyze Nitrogen Source Dependent Virulence of Candida albicans
Source: PLoS One. 2011 Nov 14;6(11):e27434. doi: 10.1371/journal.pone.0027434 (PMC3215725; doi:10.1371/journal.pone.0027434)
Supplement: Table S1 — Percent average survival for all infections shown in this study. (DOC) [file pone.0027434.s003.doc]

| **Table S1:** Percent average survival*^a^* for all infections shown in this study | | | | | |
| --- | --- | --- | --- | --- | --- |
| **Infection with:** | **day 1** | **day 2** | **day 3** | **day 4** | **day 5** |
| **Figure 1B** |  |  |  |  |  |
| ***C. albicans* (PMRCA18)**  **(10,000 cells/ µL)***^b^* | 67.2±3.7 | 54.9±3.7 | 49.8±3.4 | 46.6±3.5 | 40.6±3.4 |
| ***C. albicans* (PMRCA18)**  **(1000 cells/ µL)** | 83.3±2.3 | 77.8±3.3 | 74.0±3.5 | 71.9±3.9 | 66.3±3.9 |
| ***C. albicans* (PMRCA18)**  **(100 cells/ µL)** | 81.1±3.5 | 77.2±3.9 | 73.1±4.1 | 71.6±4.1 | 69.8±4.2 |
| ***C. albicans* (PMRCA18)**  **(10 cells/ µL)** | 85.1±2.1 | 81.2±2.6 | 77.1±2.8 | 74.0±2.8 | 69.1±3.0 |
| ***C. albicans* (PMRCA18)**  **(1 cell/ µL)** | 93.0±1.7 | 89.2±1.9 | 87.0±1.9 | 83.0±2.0 | 79.0±2.4 |
| **Figure 1C** |  |  |  |  |  |
| **PBS** | 90.6±2.9 | 88.6±3.4 | 86.4±3.7 | 84.4±4.0 | 81.6±4.2 |
| ***S. cerevisiae* (KRY001)** | 91.0±2.2 | 87.4±2.5 | 85.4±2.5 | 83.8±2.5 | 80.4±2.6 |
| ***C. albicans* (PMRCA18)** | 70.2±7.1 | 56.2±9.3 | 52.6±9.2 | 49.0±9.4 | 45.4±8.9 |
| **heat-killed *C. albicans* (PMRCA18)** | 91±1.2 | 90.2±1.3 | 89.6±1.5 | 87.8±1.5 | 86±2.1 |
| **Figure 1D** |  |  |  |  |  |
| **OrR^BSC^ PBS** | 91.2±2.6 | 84.0±3.0 | 79.6±3.4 | 76.5±3.8 | 73.4±4.3 |
| **OrR^BSC^ *S. cerevisiae* (KRY001)** | 81.8±3.1 | 75.0±3.1 | 71.0±3.7 | 66.8±4.0 | 64.2±4.2 |
| **OrR^BSC^ *C. albicans* (PMRCA18)** | 64.2±5.5 | 47.3±4.2 | 42.1±4.0 | 39.5±4.1 | 36.7±3.8 |
| **CantonS PBS** | 87.1±2.7 | 75.8±3.6 | 68.5±3.8 | 64.1±4.2 | 59.8±4.2 |
| **CantonS *S. cerevisiae* (KRY001)** | 81.8±3.0 | 65.1±3.2 | 58.9±2.8 | 54.8±2.8 | 51.6±3.0 |
| **CantonS *C. albicans* (PMRCA18)** | 54.2±5.2 | 33.5±4.1 | 26.7±3.0 | 21.7±2.7 | 19.8±2.6 |
| **Figure 1E** |  |  |  |  |  |
| ***cph1Δ/efg1Δ* (HLC54)** | 85.0±5.1 | 79.2±5.4 | 76.0±5.5 | 74.2±5.3 | 70.6±5.2 |
| ***csh3Δ* (PMRCA12)** | 84.4±2.5 | 77.0±3.6 | 72.5±4.5 | 69.9±4.7 | 67.5±4.7 |
| **WT *C. albicans* (SC5314)** | 64.2±6.5 | 55.2±6.0 | 49.6±7.0 | 46.4±7.5 | 45±7.3 |
| ***sap2Δ* (SAP2MS4B)** | 79.8±5.0 | 71.4±6.0 | 66.2±6.0 | 63.2±5.8 | 62.4±5.9 |
| **Figure 4A** |  |  |  |  |  |
| ***stp1Δ* (PMRCA59)** | 74.4±7.7 | 67.4±9.4 | 65.0±9.2 | 63.2±9.1 | 60.8±58.8 |
| ***stp2Δ (PMRCA57)*** | 70.5±4.8 | 48.9±3.6 | 44.0±4.5 | 39.1±5.3 | 34.3±5.2 |
| ***stp1Δ stp2Δ* (PMRCA94)** | 81.6±6.9 | 73.2±8.0 | 69.2±7.8 | 68.8±8.1 | 65.0±8.0 |
| ***stp1Δ/STP1 stp2Δ* (PMRCA95)** | 75.0±4.8 | 66.2±5.8 | 61.8±5.2 | 56.8±4.4 | 53.6±4.3 |
| ***stp1Δ/STP1** (PMRCA60)** | 66.6±7.3 | 56.4±7.7 | 49.4±7.0 | 46.6±7.4 | 42.4±7.0 |
| **Figure 4C** |  |  |  |  |  |
| **WT *C. albicans* (PMRCA18)** | 78.5±6.4 | 55.3±6.4 | 49.7±6.0 | 44.1±5.8 | 39.1±5.6 |
| ***stp1Δ* (PMRCA59)** | 95±1.2 | 87.4±3.1 | 82.2±3.8 | 76.6±4.5 | 72.0±5.5 |
| ***ssy1Δ* (YJA64)** | 86.8±5.8 | 78.5±7.2 | 74±8.2 | 70.8±8.2 | 68.5±8.1 |
| ***ssy5Δ* (YJA53)** | 87.5±4.2 | 76±7.1 | 70.8±8.1 | 66±8.5 | 61.8±8.3 |
| **Figure 4E** |  |  |  |  |  |
| ***Tl^632^/Tl^(1-RXA)^*PBS** | 98.3±1.7 | 95.1±0.1 | 90.2±2.7 | 83.0±2.6 | 78.2±4.5 |
| ***Tl^632^/Tl^(1-RXA)^S. cerevisiae* (KRY001)** | 97.5±2.5 | 92.6±2.6 | 85.4±0.4 | 83.0±2.0 | 73.1±3.1 |
| ***Tl^632^/Tl^(1-RXA)^C. albicans* (PMRCA18)(10,000 cells/µL)** | 27.7±3.6 | 17.4±2.9 | 6.8±2.7 | 5.2±1.9 | 4.3±1.8 |
| ***Tl^632^/Tl^(1-RXA)^stp1Δ* (PMRCA59)(10,000 cells/µL)** | 28.6±6.2 | 16.7±4.8 | 9.5±2.9 | 7.3±1.9 | 6.0±0.7 |
| ***Tl^632^/Tl^(1-RXA)^C. albicans* (PMRCA18)(1000 cells/µL)** | 83.3 | 30.6 | 22.2 | 16.7 | 11.1 |
| ***Tl^632^/Tl^(1-RXA)^stp1Δ* (PMRCA59)(1000 cells/µL)** | 75 | 25 | 14.3 | 7.1 | 3.6 |
| ***Tl^632^/Tl^(1-RXA)^C. albicans* (PMRCA18)(100 cells/µL)** | 82.1 | 56.4 | 38.4 | 33.3 | 28.2 |
| ***Tl^632^/Tl^(1-RXA)^stp1Δ* (PMRCA59)(100 cells/µL)** | 100 | 63.2 | 42.1 | 36.8 | 26.3 |

*^a^* values listed are means ± SEM (at least four samples per group).

*^b^* OrR^YE^ was the strain infected unless otherwise indicated.
