# Supplementary material for: Wild-Type Drosophila melanogaster as a Model Host to Analyze Nitrogen Source Dependent Virulence of Candida albicans
Source: PLoS One. 2011 Nov 14;6(11):e27434. doi: 10.1371/journal.pone.0027434 (PMC3215725; doi:10.1371/journal.pone.0027434)
Supplement: Table S2 — Fungal strains used in this study. (DOC) [file pone.0027434.s004.doc]

| **Table S2:** Fungal strains used in this study. | | |
| --- | --- | --- |
| **Name** | **Genotype** | **Reference** |
| *Candida albicans* CAI4 background derived strains | | |
| HLC54 | *cph1::hisG/cph1::hisG efg1::hisG/efg1::hisG-URA3 ura3::imm434/ura3::imm434* | [20] |
| PMRCA12 | *csh3Δ3/csh3Δ3 ura3::imm434/URA3* | [11] |
| PMRCA18 | *ura3::imm434/URA3* | [11] |
| PMRCA57 | *stp2Δ4*::*dpl200-URA3/stp2Δ2*::*CaNAT1/stp2Δ5*::*MPA^r^ ura3Δ*::*imm434/ura3Δ*::*imm434* | [3] |
| PMRCA59 | *stp1Δ1/stp1Δ1 ura3Δ::imm434/URA3* | [3] |
| PMRCA60 | *stp1Δ1/STP1Δ62 ura3Δ::imm434/URA3* | [3] |
| PMRCA94 | *stp1Δ1/stp1Δ1 stp2Δ5::MPA^r^/stp2Δ2::CaNAT1/stp2Δ4::dpl200 ura3Δ::imm434/URA3* | [3] |
| PMRCA95 | *stp1Δ1/stp1Δ1::STP1-URA3 stp2Δ5::MPA^r^/stp2Δ2::CaNAT1/stp2Δ4::dpl200 ura3::imm434/ura3::imm434* | [3] |
| YJA53 | *ssy5Δ::FRT/ssy5Δ::FRT ura3::imm434/URA3* | This work |
| YJA64 | *ssy1Δ::FRT/ssy1Δ::FRT ura3::imm434/URA3* | This work |
| *Candida albicans* SC5314 background derived strains | | |
| SC5314 | prototrophic wild-type | [39] |
| SAP2MS4B | *sap2Δ::FRT/sap2Δ::FRT* | [21] |
| *Saccharomyces cerevisiae* Σ1278b background | | |
| KRY001 | *MATα/MATa ura 3-52/URA3* | This work |
