# Supplementary material for: Wild-Type Drosophila melanogaster as a Model Host to Analyze Nitrogen Source Dependent Virulence of Candida albicans
Source: PLoS One. 2011 Nov 14;6(11):e27434. doi: 10.1371/journal.pone.0027434 (PMC3215725; doi:10.1371/journal.pone.0027434)
Supplement: Table S3 — Pair-wise statistical analysis of survival curves at day 3 post-infection. (DOC) [file pone.0027434.s005.doc]

| **Table S3:** Pair-wise statistical analysis of survival curves at day 3 post-infection. | | |
| --- | --- | --- |
| **Fungal strain** | **PBS vs. strain (**p-value) | ***C. albicans* WT vs. strain**  (p-value) |
| ***C. albicans*** |  |  |
| WT (PMRCA18) | < 0.001 |  |
| WT (PMRCA18; 1000 cells/µl) | 0.007 | < 0.001 |
| WT (PMRCA18; 100 cells/µl) | 0.005 | < 0.001 |
| WT (PMRCA18; 10 cells/µl) | 0.029 | < 0.001 |
| WT (PMRCA18; 1 cell/µl) | 0.886 | < 0.001 |
| Heat killed WT (PMRCA18) | 0.995 | < 0.001 |
| PBS into OrR^BSC^ | 0.137 | < 0.001 |
| WT (PMRCA18) into OrR^BSC^ | < 0.001 | 0.263 |
| PBS into CanS | 0.002 | 0.007 |
| WT (PMRCA18) into CanS | < 0.001 | 0.001 |
| *cph1Δ efg1Δ* (HLC54) | 0.040 | 0.008 |
| *csh3Δ* (PMRCA12) | 0.008 | 0.039 |
| *sap2Δ* (SAP2MS4B)* | 0.003 | 0.05 |
| *stp1Δ* (PMRCA59)** | 0.001, 0.345 | 0.155, <0.001 |
| *stp2Δ* (PMRCA57) | <0.001 | 0.525 |
| *stp1Δ/stp2Δ* (PMRCA94) | 0.031 | 0.008 |
| *stp1Δ/stp2Δ::STP1* (PMRCA95) | <0.001 | 0.383 |
| *stp1Δ/STP1** (PMRCA60) | <0.001 | 0.624 |
| *ssy1Δ* (YJA64) | 0.007 | 0.072 |
| *ssy5Δ* (YJA53) | 0.003 | 0.137 |
| ***S. cerevisiae*** |  |  |
| WT (KRY001) | 0.391 | < 0.001 |

* *sap2Δ* *C. albicans* values were compared statistically to a wild-type strain of its background, WT (SC5314).

** was used in two different experiments displayed in Fig. 4A and 4C, respectively, where the experiments displayed in Fig. 4C (p< 0.001) were done by an improved infection protocol (see M&M)
